# Matching clinical and genetic data in pediatric patients at risk of developing cystic kidney disease

**DOI:** 10.1007/s00467-024-06548-6

**Published:** 2024-10-10

**Authors:** Valeria Bracciamà, Tiziana Vaisitti, Fiorenza Mioli, Angelo Corso Faini, Giulia Margherita Brach del Prever, Vitor Hugo Martins, Roberta Camilla, Francesca Mattozzi, Silvia Pieretti, Maria Luca, Carmelo Maria Romeo, Claudia Saglia, Martina Migliorero, Francesca Arruga, Diana Carli, Antonio Amoroso, Pietro Lonardi, Silvia Deaglio, Licia Peruzzi

**Affiliations:** 1 Immunogenetics and Transplant Biology, AOU Città della Salute e della Scienza, ERKNet Center & Department of Medical Sciences, University of Turin, Turin, Italy; 2 Nephrology Dialysis and Transplantation, ERKNet Center, Regina Margherita Children’s Hospital, Turin, Italy

**Keywords:** Cystic disease, Hyper-echogenic kidneys, Early onset, Clinical exome sequencing, Genetic heterogeneity

## Abstract

**Background:**

Cystic kidney disease is a heterogeneous group of hereditary and non-hereditary pathologic conditions, associated with the development of renal cysts. These conditions may be present both in children and adults. Cysts can even be observed already during the prenatal age, and pediatric patients with cysts need to be clinically monitored. An early clinical and genetic diagnosis is therefore mandatory for optimal patient management. The aim of this study was to perform genetic analyses in patients with echographic evidence of kidney cysts to provide an early molecular diagnosis.

**Methods:**

A cohort of 70 pediatric patients was enrolled and clinically studied at the time of first recruitment and at follow-up. Genetic testing by clinical exome sequencing was performed and a panel of genes responsible for “cystic kidneys” was analyzed to identify causative variants. Sanger validation and segregation studies were exploited for the final classification of the variants and accurate genetic counseling.

**Results:**

Data showed that 53/70 of pediatric patients referred with a clinical suspicion of cystic kidney disease presented a causative genetic variant. In a significant proportion of the cohort (24/70), evidence of hyper-echogenic/cystic kidneys was already present in the prenatal period, even in the absence of a positive family history.

**Conclusions:**

This study suggests that cystic kidney disease may develop since the very early stages of life and that screening programs based on ultrasound scans and genetic testing play a critical role in diagnosis, allowing for better clinical management and tailored genetic counseling to the family.

**Graphical Abstract:**

**Figure:**
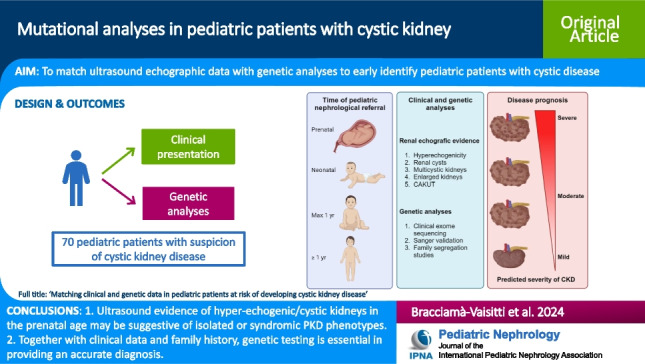
A higher resolution version of the Graphical abstract is available as Supplementary information

**Supplementary Information:**

The online version contains supplementary material available at 10.1007/s00467-024-06548-6.

## Introduction

Cystic kidney disease is an umbrella term that refers to a variety of hereditary and non-hereditary conditions, most of them characterized by prenatal onset with consequent high neonatal morbidity and mortality [1]. Early-onset cystic diseases include autosomal dominant polycystic kidney disease (ADPKD), autosomal recessive PKD (ARPKD), *HNF1B*-nephropathy, tuberous sclerosis complex (TSC), and other ciliopathies. In some instances, clinical presentation may overlap with congenital anomalies of the kidneys and of the urinary tract (CAKUT) [2].

ADPKD is the most common monogenic cause of kidney failure. Approximately 80% of patients harbor causative variants in *PKD1*, whereas *PKD2* accounts for 10–15% of the cases [3]. Of note, 5–10% of ADPKD is due to rare variants in other loci [4]. Several genetic and non-genetic factors can modulate cyst formation and disease progression, including biallelic variants either in PKD or in other genes linked to PKD, variants in modifier genes, and environmental factors such as acute kidney injury (AKI) [5–7]. Given that a significant proportion of patients remain a-/pauci-symptomatic until adulthood, ADPKD is typically considered a disease of adults. However, in childhood, comorbidities or early signs of disease may be underdiagnosed, unless actively investigated. In addition, some patients, defined as very early-onset, develop renal hyper-echogenicity or cysts in the prenatal period or at birth, with a generally more rapid disease progression, compared to patients diagnosed much later in life [8, 9]. Other patients, classified as early-onset (EO), present the clinical phenotype during childhood (2–14 years) [6]. The risk for prenatal disease manifestation is increased in families with previous cases of prenatal-onset ADPKD [6]. Interestingly, a significant proportion of patients (22–25%) with ADPKD have no family history of the disease, suggesting that de novo variants occur at a considerable rate [10]. Based on this evidence, increased consciousness of prenatal/neonatal development of cystic disease, as well as a prompt clinical and diagnostic framework are necessary for the management of children at risk of or diagnosed with cystic kidney disease. However, a precise diagnosis of EO cystic kidney disease may be complicated because of high phenotypic overlap and extreme heterogeneity [9]. Genetic testing is essential in the early identification of the specific molecular defects and in formulating the correct diagnosis, allowing for better clinical management of the patient and a correct estimate of the risk of recurrence in the family of affected individuals.

This work aims at providing a snapshot of the clinical features and mutational burden of a cohort of pediatric patients, recruited at a single Institution, and referred for genetic testing with clinical suspicion of cystic disease of the kidney.

## Methods

### Patient recruitment

Seventy pediatric patients (all < 18 years at recruitment) with kidney hyper-echogenicity or cysts at prenatal or early-childhood ultrasound were followed from 2003 to 2023 by the Pediatric Nephrology, Dialysis and Transplantation Unit of the Città della Salute e della Scienza Hospital (Turin, Italy). They were referred to the Immunogenetics and Transplant Biology Service of the same hospital for genetic testing. Written informed consent for genetic analysis and research was obtained from the patients’ parents, in compliance with National Ethical Regulations. The study was approved by the Institutional Ethics Committee (#42270, dated April 12, 2022).

### Sample preparation, sequencing, and bioinformatic analyses

DNA extraction from peripheral blood, library preparation, clinical exome sequencing (CES), and data analyses were performed as previously reported [11]. Data were hierarchically analyzed based on in silico gene lists: firstly, genes associated with polycystic kidney (*PKD1, PKD2*, *PKHD1*, and *IFT140*). In the case of a negative analysis, an extension to genes associated with cystic kidney diseases was performed (~100 genes; Table 1). Variants were classified according to the American College of Medical Genetics and Genomics (ACMG) criteria [12].

**Table 1:** Patients were analyzed for polycystic kidney associated genes, and if negative, analysis was extended to cystic disease associated genes

**Polycystic kidney**	*PKD1, PKD2, PKHD1, IFT140*
**Cystic disease**	*AHI1, ALG5, ALG8, ALG9, ALMS1, ANKS6, ARL13B, ARL6, B9D1, B9D2, BBS1, BBS10, BBS12, BBS2, BBS4, BBS5, BBS7, BBS9, C2CD3, C5orf42, CC2D2A, CCDC28B, CCND1, CDC73, CEP120, CEP164, CEP290, CEP41, CEP83, COL4A1, COL4A3, COL4A4, COL4A5, COL4A6, CRB2, CSPP1, CYS1, DDX59, DHCR7, DLG5, DYNC2H1, FAN1, FLCN, GLIS2, HNF1B, HYLS1, ICK, IFT122, IFT140, IFT172, IFT27, IFT43, INVS, IQCB1, KIF14, KIF7, LRP5, LZTFL1, MKKS, MKS1, MUC1, NEK1, NEK8, NOTCH2, NPHP1, NPHP3, NPHP4, OFD1, PAX2, PIBF1, PIGT, PMM2, PRKCSH, RAD51C, RPGRIP1L, SCLT1, SDCCAG8, SEC63, SLC41A1, TCTN1, TCTN2, TCTN3, TMEM138, TMEM216, TMEM231, TMEM237, TMEM67, TRAF3IP1, TSC1, TSC2, TTC21B, TTC8, TULP3, UMOD, VHL, WDPCP, WDR19, WDR35, WDR60, XPNPEP3, ZNF423*

### Sanger sequencing and multiplex ligation-dependent probe amplification (MLPA)

Sanger sequencing and MLPA analyses were performed to validate identified variants and for family segregation studies, as previously reported [13, 14]. In case of either inconclusive/negative CES analysis, or copy number variant (CNV) identification by CES, MLPA analyses were performed using SALSA MLPA P351 *PKD1* and P352 *PKD1*–*PKD2* probe mix, while *HNF1B* CNVs were validated by P241 MODY Mix 1 (MRC-Holland, Amsterdam, NL).

## Results

### Main features of the study cohort

We performed genetic testing on 70 unrelated patients referred to the Immunogenetics and Transplant Biology Service because of ultrasound early evidence of hyper-echogenic and/or cystic kidneys. Patients were mostly males (46, 65.7%) of Caucasian origin (64, 91%). At the time of genetic testing, 38 patients (54%) were between 3 and 14 years old, while 23 (33%) were in the 0–2 age range.

### Description of the cohort at the time of pediatric nephrology referral

Twenty-two patients (31.4%) were referred in the prenatal period: 7 presented with bilateral renal hyper-echogenicity, 8 with renal cysts, 2 had a single multicystic kidney, 1 showed agenesis of the right kidney and cysts in the left one, and the remaining 4 patients presented respectively with enlarged kidneys, kidney dysplasia, anhydramnios, and dilation of the urinary tract. Among them, 5 patients underwent prenatal genetic testing, while the remaining 17 were diagnosed after birth.

Eight patients (11.4%) were referred to the pediatric nephrologist in the first 30 days of life for (i) AKI (*n* = 3), (ii) enlarged abdomen and palpable kidneys (*n* = 2), (iii) a suspicion of tubular disease (*n* = 1), and (iv) a clinical suspicion of ARPKD (*n* = 1). The remaining patient was referred after a kidney ultrasound was performed as part of a neonatal screening program to detect CAKUT.

Fifteen children (21.4%) were referred in the first year of life for (i) familial screening (*n* = 3), (ii) abdominal enlargement with a suspected tumor mass (*n* = 5), or (iii) after ultrasound performed following a urinary tract infection or CAKUT suspicion (*n* = 4). The remaining 3 patients were referred after kidney ultrasound as part of the screening program for CAKUT.

Finally, 25 out of 70 children (35.7%) were referred between 18 months and 15 years, median 6.7 years, as none of them presented with prenatal evidence of cystic disease or enlarged abdomen. Within this subgroup of patients, cysts were highlighted following ultrasounds performed for trauma or abdominal pain of unknown reasons (*n* = 4) and familial screening in asymptomatic subjects (*n* = 11), following urinary tract infection, macroscopic hematuria, or other reasons for ultrasound analysis (*n* = 10).

**Fig. 1:**
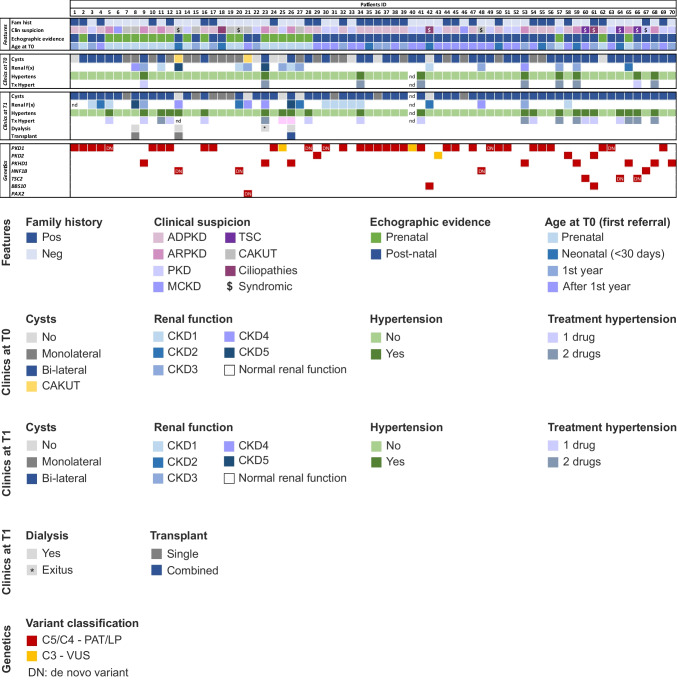
Main clinical features of the study cohort and identified causative variants. General features (family history, clinical suspicion, pre-term birth, and age at first visit), clinical features (presence of cysts, enlarged abdomen, kidney function, hypertension, and treatment of hypertension) at the time of the first pediatric nephrology referral (T0) and at the time of follow up (T1), and genetic results for each patient of the study cohort are reported. Fam hist, family history; Clin suspicion, clinical suspicion; Enlarge abd, enlarged abdomen; Hypertens, hypertension; Tx hypert, treatment of hypertension; Pos, positive; Neg, negative; ADPKD, autosomal dominant polycystic kidney disease; ARPKD, autosomal recessive polycystic kidney disease; PKD, polycystic kidney disease; MCKD, medullary cystic kidney disease; TSC, tuberous sclerosis complex; Cil, ciliopathies; Mono, mono-lateral cysts; Bi-lat, bi-lateral cysts; $, syndromic features; PAT, pathogenic; LP, likely pathogenic; VUS, variant of unknown significance; DN, de novo variants. CKD1, 90 ml/min/1.73 m^2^; CKD2, 60–89 ml/min/1.73 m^2^; CKD3, 30–59 ml/min/1.73 m^2^; CKD4, 15–29 ml/min/1.73 m^2^; CKD5, < 15 ml/min/1.73 m^2^

Of note, 9 children (12.8%) presented with clinical signs suggestive of a syndromic disease: 3 patients were referred for TSC (2 at neonatal age and 1 at 2 years of age) and 6 presented with kidney cysts with relevant neurological signs (Fig. 1).

### Correlation between clinical suspicion and genetic findings

**Fig. 2:**
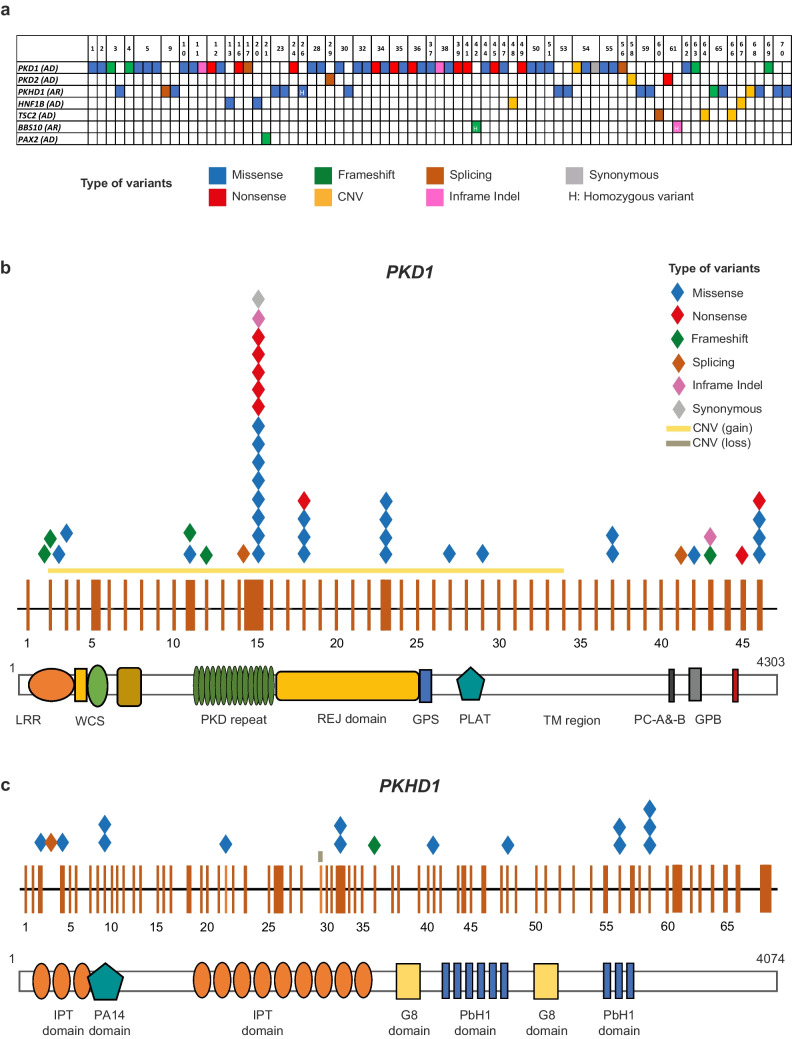
Scheme of causative variants within the cohort and representation of the most recurrently mutated genes. Summary of the identified causative variants for each patient (Pts) based on their nature (type of variant indicated as a color code), gene involved, and its mode of inheritance. AD, autosomal dominant; AR, autosomal recessive; CNV, copy number variant; H, homozygosity (**a**). Diagram of gene and protein structure of *PKD1* (**b**) and *PKHD1* (**c**) as the most recurrently mutated genes in the cohort. Exons are shown as orange rectangles, and the main protein functional domains are depicted. Color-code diamonds represent the identified causative variants. LRR, leucine-rich repeats; WCS, cell-wall and stress-response component; PKD, polycystic kidney disease; REJ, receptor for egg-jelly; GPS, G-protein-coupled receptor protein; PLAT, polycystin-1, lipoxygenase, alpha toxin; TM, transmembrane; PC-A & -B, polycystin homologous motif A & B; GPB, G-protein binding; IPT, immunoglobulin-like-plexin-transcription factor; PbH1, parallel ß-helix 1; DD, dimerization domain

Overall, we identified 77 causative variants: most of them mapped in *PKD1* (*n* = 47, 61.0%), followed by *PKHD1* (*n* = 17, 22.1%) and *HNF1B* (*n* = 4, 5.2%). *PKD2* showed a lower rate of alterations, presenting only in three variants (*n* = 3, 3.9%). *PKD1* was enriched for missense (*n* = 26, 55.3%) and nonsense variants (*n* = 11, 23.4%), with an overall rate of non-truncating vs. truncating variants of 68.2 vs. 31.8%. On the contrary, *PKHD1* presented mainly missense variants (*n* = 14, 82.3%; Fig. 2a). In 2/4 patients with a genetic diagnosis of *HNF1B*-nephropathy, a CNV (loss) involving the whole gene was identified (Fig. 2a).

In the five prenatal tests, causative variants were identified in *PKD1* (two patients, #16 and #62) and *TSC2* (#60). Of note, one patient with a *PKD1* variant was affected also by *GJC2* leukodystrophy. The other two patients remained undiagnosed (Fig. 1).

In the 26 children (37.1%) referred with a clinical suspicion of ADPKD, genetic analysis was always diagnostic with pathogenic variants identified in *PKD1* (*n* = 24, 92.3%) or *PKD2* (*n* = 2, 7.7%). Of 9 patients (12.9%) referred with a clinical suspicion of ARPKD, 7 (77.8%) received molecular confirmation with one biallelic variant (*n* = 1) and two compound heterozygous *PKHD1* variants (*n* = 6). Interestingly, in the two remaining patients (22.2%; #5 and #11), genetic testing showed two causative in trans *PKD1* variants (Fig. 2a).

Out of 9 patients with cystic kidneys and CAKUT, genetic variants were identified in 6 (66.7%). Based on the family study, 4 patients (44.4%) received a molecular diagnosis with causative variants in *HNF1B* (*n* = 3, #13, 20, 48) and *PAX2* (*n* = 1, #21), while for the rest, it was non-diagnostic (#7, 8, 15, 19, and 27).

Of the 3 patients (4.3%) with a suspected ciliopathy, one (#42) was genetically diagnosed with Bardet–Biedl syndrome, as she presented with a biallelic *BBS10* variant. In one patient (#61), besides a biallelic *BBS10* variant, a pathogenic *PKD2* variant was identified leading to a double genetic diagnosis (Bardet–Biedl syndrome and ADPKD; Fig. 2a). Genetic testing in the remaining patient was non-diagnostic.

Three children, referred with a clinical diagnosis of TSC, obtained genetic confirmation with pathogenic *TSC2* variants. In two patients (#64, 66), family segregation showed a de novo phenotype (Figs. 1 and 2a).

In the last 18 cases, kidney ultrasound highlighted isolated cysts that were difficult to classify based solely on imaging data. Genetic analysis identified compatible variants in 16 patients (88.9%) and family segregation studies confirmed a genetic diagnosis in 9 of them (56.2%): ADPKD–*PKD1* (7), *HNF1B*-related disease (1), and ARPKD (1). Three patients carried VUS variants, 2 in *PKD1* (#25, 40) and 1 in *PKD2* (#43), and in the last 4 cases, variants were excluded due to inconsistent segregation (#22, 31, 52, 57). Two patients received a negative genetic test (Figs. 1 and 2a).

Neither of the 2 children with a clinical suspicion of medullary cystic kidney disease (MCKD) (#6, 46) had a conclusive genetic test.

### Correlation between positive PKD family history and genetic findings

Twenty-nine patients (41.4%) presented with a positive family history of PKD, and four patients had relatives with additional clinical features, including cerebral hemorrhage and acute myocardial infarction. Remarkably, causative genetic variants were identified in all of them: 28 (96.5%) were diagnosed with ADPKD and one with *HNF1B*-related disease. The remaining 41 patients (58.6%) had a negative family history, and causative genetic variants were identified in 24 of them (58.5%, Fig. 1).

### Distribution of the genetic variants

*PKD1* showed four mutational hot spots located in exons 15, 18, 23, and 46 that hosted 25 out of the 40 variants (62.5%), with consequent involvement of the PKD repeat, REJ, and C-terminal domains (Fig. 2b). Of note, exon 15 of *PKD1* hosted most nonsense variants. On the contrary, *PKHD1* did not show any hot spot region, as variants mapped in different exons along the whole gene (Fig. 2c). Finally, for *HNF1B,* 50% of the identified variants were represented by a CNV that involved the entire gene.

Among the 53 patients with genetic diagnosis, 22 (41.5%) carried multiple variants either in the same gene or in different cystogenes. Specifically, 12 patients had multiple *PKD1* variants (50% confirmed in trans), 2 carried an additional *PKHD1* variant besides the one in *PKD1*, and 1 patient with a *BBS10* variant carried also a *PKD2* variant. The remaining 7 patients were characterized by variants in *PKHD1*.

### Nephrology follow-up and genetic matching

**Fig. 3:**
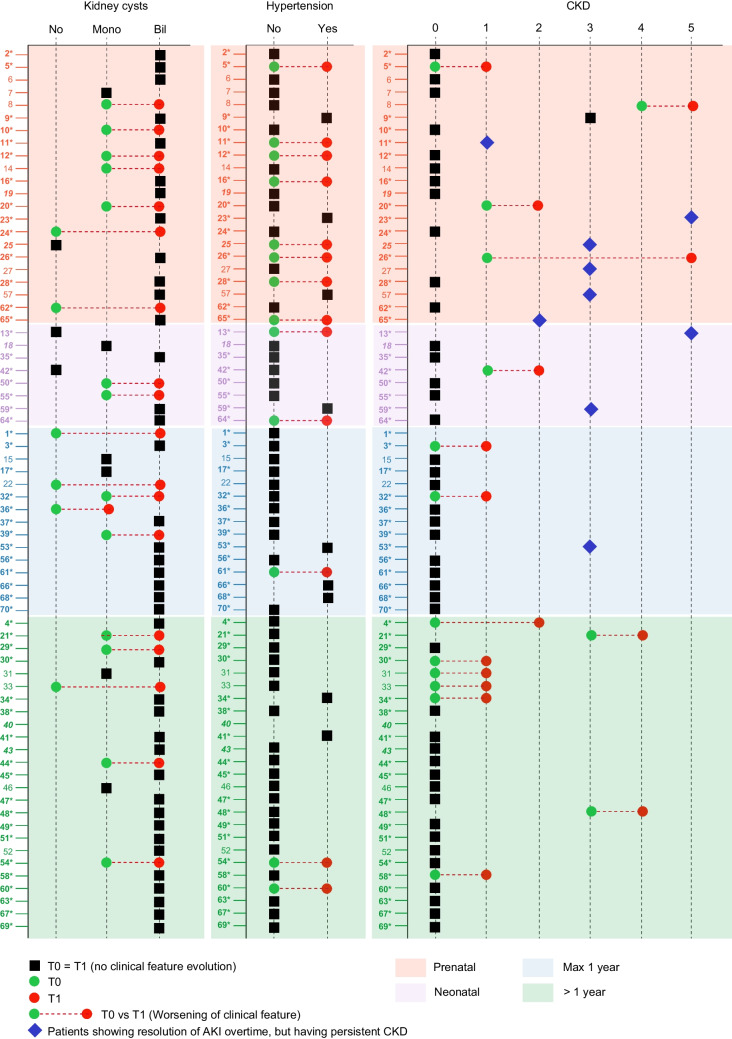
Evolution of disease features between first referral and follow-up time points. Analysis of kidney cysts, hypertension, and renal function (CKD) at the time of first referral (T0) and follow-up (T1) in the study cohort divided based on first referral: prenatal, neonatal, maximum 1 year of life, and older than 1 year old. For cysts, patients were reported with no cysts (No), monolateral (Mono), and bilateral cysts (Bil). For hypertension, patients were reported as negative (No) or positive (Yes). Regarding renal function, patients were reported as with a normal function (CKD0, significantly higher than 90 ml/min/1.73 m^2^; CKD1, 90 ml/min/1.73 m^2^) or a compromised function (CKD2, 60–89 ml/min/1.73 m^2^; CKD3, 30–59 ml/min/1.73 m^2^; CKD4, 15–29 ml/min/1.73 m^2^; CKD5, < 15 ml/min/1.73 m^2^). Blue diamonds indicate those patients showing mild or severe AKI at birth or at neonatal age with progressive resolution of the acute phase, but still showing CKD. In bold with an asterisk are reported patients with a positive genetic test, while in bold italics are those patients with VUS variants potentially causative of the phenotype based on segregation studies

The median follow-up of the whole cohort is 4 years (2 months–18 years). Of the 58 (82.8%) patients who presented with kidney cysts at the first ultrasound, 30 had < 10 cysts, 28 had > 10 cysts, and bilateral cysts were present in ~50% of the patients. Five children also presented with liver cysts, while 12 patients had bilaterally enlarged kidneys. Only two children with ARPKD required nephrectomy, one at 4 months after birth and the other when he was 6 (Fig. 3).

At follow-up, 12 patients with mono-lateral cysts at first observation developed bilateral cysts that increased also in number. Of these, 10/12 had a positive genetic test: 8 showed C4/C5 variants in *PKD1*, 1 in *PKD2,* and the remaining one in *HNF1B.* Moreover, 4 children, who showed normal liver at the first ultrasound, developed liver cysts during follow-up. All of them were characterized by genetic variants mapping in *PKD1* (*n* = 1), *PKHD1* (*n* = 2), and *TSC2* (*n* = 1; Fig. 3).

At first observation, kidney function was normal in 56 (80.0%) patients while the remaining cohort was distributed as follows: 4 in CKD1 (eGFR ~90 ml/min/1.73 m^2^), 1 in CKD2 (eGFR 60–89 ml/min/1.73 m^2^), 7 in CKD3 (eGFR 30–59 ml/min/1.73 m^2^), 1 in CKD4 (eGFR < 30 ml/min/1.73 m^2^), and 2 patients had AKI on CKD5 (eGFR < 15 ml/min/1.73 m^2^).

During follow-up, six children reached a stage of kidney failure higher than CKD2. Of the four children who required dialysis, two had a genetic diagnosis of ARPKD (one at 6 months, and the other at 8 years of age), one had a diagnosis of HNF1B-related disease and ARPKD and was 6 months old and the last one was 6 years old and had an inconclusive genetic diagnosis. Two children received a kidney transplant at 5.6 and 6.9 years, respectively, and another one with ARPKD received a liver–kidney transplant at 12. One child affected by ARPKD died at 7 months due to severe lung dysplasia and multiorgan failure (Figs. 1 and 3). Even though the cohort is limited, these data suggest, as somewhat expected, that the worst and most rapid progression of disease was associated with ARPKD, *HNF1B*-related disease, and tuberous sclerosis.

In 9 cases, mostly with prenatal evidence of severe kidney derangement, an improvement of kidney function during follow-up was observed, due to resolution of perinatal AKI or kidney function maturation. In 1 case, the first observation was of AKI (6 months) on a previously undetected ARPKD and residual CKD (Fig. 3).

Arterial hypertension was diagnosed in 9/70 patients, including a newborn. Seven infants (< 1 year old) required two drugs to reach optimal control of blood pressure. Among these patients, 4 had an ARPKD, 1 ADPKD–*PKD1*, and 1 biallelic ADPKD, and the remaining patient, diagnosed with Turner syndrome, had a negative genetic test for PKD genes. Interestingly, Turner patients can present with kidney cysts in the absence of genetic variants in PKD-related genes [15]. Lastly, 1 child (11 years old) received treatment for hypertension since the age of 5, in the absence of a precise diagnosis, and proved to be affected by ADPKD (Figs. 1 and 3).

At follow-up, 22 children displayed arterial hypertension requiring therapy in the pediatric age (median age of 9 months; 1 month–12 years; Fig. 3). Among the children displaying hypertension within the first year of life (*n* = 12), the majority was diagnosed with ARPKD (*n* = 6), followed by biallelic ADPKD, TSC–PKD1 contiguous syndrome (*n* = 2, respectively), ADPKD (*n* = 1), and HNF1B-related disorder (*n* = 1). Two children experienced cerebral hemorrhage due to arteriovenous malformation of the cerebral artery at 14 months and at 12 years, both affected by bi-allelic ADPKD.

## Discussion

This work was carried out with the aim of analyzing a retrospective cohort of 70 pediatric patients followed because of cystic kidneys by the Pediatric Nephrology Unit between 2003 and 2023. This cohort includes children with a positive ultrasound for cystic, hyperechogenic, or enlarged kidneys during the prenatal age or diagnosis of cystic disease in the post-natal age. All patients underwent genetic testing, and whenever possible, variants were validated by family segregation studies (74.3%; 52/70). By matching clinical and genetic data, a few points are worthy to be highlighted, distinguishing the prenatal and post-natal cohorts.

First, 22 patients presented with hyper-echogenic/cystic kidneys in the prenatal age, as detected by routine ultrasound screening. Importantly, the majority of these patients have ADPKD and not ARPKD. Of them, 14 (63.6%) presented causative variants, and it is interesting to note that 8 ADPKD patients with abnormal prenatal ultrasound findings carried variants in exon 15 and 46, two well-known mutational hot spots in the *PKD1* gene, and 2 carried two in trans variants in the same gene. These results underline the need to carefully evaluate ultrasound images during pregnancy, checking for kidney hyper-echogenicity and/or cysts, as they may be symptomatic of isolated or syndromic phenotypes [16]. Moreover, they suggest that careful screening is critical for the early identification of patients with potential cystic kidney disease.

Secondly, when looking at the post-natal cohort, 22/48 presented with a positive family history, and all were genetically diagnosed with causative variants mainly involving *PKD1* (18 patients), *PKD2* (3 patients), and *HNF1B* (1 patient). Interestingly, five of them presented with two variants *in trans* in *PKD1* and exon 15 hosted a significant proportion (34.6%) of the overall identified variants. The remaining post-natal patients (26/48; 66.7%) were referred following ultrasound performed for non-cystic-related reasons (e.g., trauma and infections). Within this cohort, 17/26 (65.4%) were genetically solved with variants mapping in different PKD-related genes. At variance with the above subsets of patients, when considering the *PKD1* gene, no variant enrichment in hot spot regions was observed.

A third noteworthy point of discussion is the need for a careful evaluation of the clinical features of the patients, together with the evaluation of the family history, to make a correct diagnosis that will be helpful even for the application of a genetic test. This consideration comes from the observation that within the 26 patients referred with a clinical suspicion of ADPKD, all (i) presented with a positive family history, (ii) already had bilateral cysts at the time of first nephrology referral or developed them during the follow-up, and (iii) were characterized by a high number of cysts. All these patients were diagnosed with pathogenic variants in *PKD1* and *PKD2*, underlining a strong correlation between genetic alterations and clinical presentation.

At the same time, patients that turned out to be negative from the genetic standpoint with no pathogenetic variants detected were all characterized by the absence of a family history of cystic diseases and from the clinical standpoint, presented with (i) isolated cysts, (ii) cysts within a single kidney, or (iii) CAKUT and (iv) absence of disease evolution, including an increase in cyst number over time.

Even though our cohort is somewhat limited in the number of patients, making it difficult to derive further clinical and genetic correlations, the abovementioned observations may represent points that open up further considerations and analyses, especially in larger cohorts or cohorts with a longer time of follow-up. It is our belief that the results obtained within this study underline the need for careful clinical evaluations in pediatric patients since the prenatal age and the early diagnosis, even through genetic analyses, is critical in specific subsets of patients.

A relevant point to be underlined is the development of clinical complications. Half of the children diagnosed prenatally developed arterial hypertension during the first years of life and required treatment. Furthermore, when compared to patients referred to the pediatric nephrologist after the first year of life, the prenatal subset is characterized by a worse kidney function (Fig. 3). This data should alert the pediatrician to the need for a genetic test. Indeed, > 50% of these patients presented with a positive genetic test in the absence of a family history of cystic kidney disease, and most of them showed pathogenic variants causative of ADPKD, followed by ARPKD and *HNF1B*-nephropathy. Moreover, 4 ADPKD patients had at least one family member who suffered from vascular events, including ruptured aortic aneurysm and intracranial hemorrhage. Nonetheless being rare, cerebrovascular events are the most dangerous complications resulting in morbidity and mortality in PKD patients. Overall, the prevalence of intracranial aneurysms is fivefold higher than in the general population and is further increased in patients with a positive family history of aneurysms or subarachnoid hemorrhage [17, 18]. Even though there are no current guidelines for pediatric patients with ADPKD to screen for asymptomatic aneurysms, these data suggest this subset of patients needs to be carefully monitored, as discussed and proposed also by a recent paper by Walker and colleagues [19].

Currently, there are no medications available to delay cyst formation for neonatal PKD, but a correct diagnosis is crucial to manage hypertension and to prolong renal function. The vasopressin receptor 2 antagonist tolvaptan, an FDA-EMA-AIFA–approved therapeutic agent for ADPKD, can currently be administered exclusively to adult patients [20], even though its efficacy in pediatric patients has been demonstrated [21, 22]. Although ADPKD is a progressive disease, early intervention has proven to be important and beneficial in clinical studies. Children with ADPKD show a higher rate of total kidney volume (TKV) increase compared to adults, which is confirmed to be a marker of progression of ADPKD [23]. Models of the evolution of TKV suggest that initiation of tolvaptan at early stages of the disease (eGFR > 60 mL/min/1.73 m^2^) exerts a more significant effect on the preservation of kidney function and improves the slowdown of cyst formation when compared to treatment initiated at later stages [21].

**Fig. 4:**
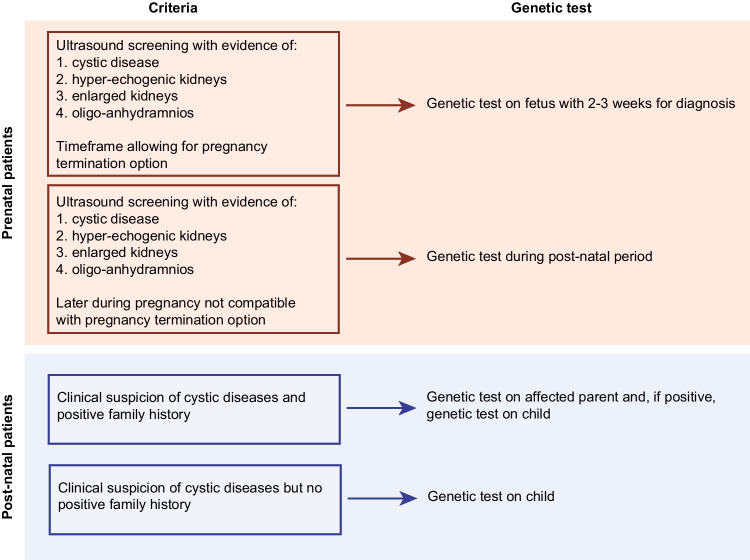
Proposed genetic diagnostic workflow in pediatric prenatal and post-natal patients. Scheme of a potential diagnostic workflow of pediatric patients, distinguishing between prenatal and post-natal subsets. Based on clinical criteria and time of clinical suspicion, a genetic test may be performed during the prenatal age or after birth on children or on affected parents in case of positive family history. All figures were generated using Adobe Illustrator

Finally, based on the results of this work, we can propose a flowchart to be adopted in the diagnostic workflow of pediatric patients, distinguishing between prenatal and post-natal patients. In the presence of cystic/hyper-echogenic/enlarged kidneys identified by ultrasound screening during the prenatal age, often in the presence of oligo-anhydramnios, and in the timeframe allowing for pregnancy termination option, the suggestion is to proceed with genetic testing that must ensure results within 2–3 weeks. In case of clinical evidence later in the pregnancy, genetic testing should be postponed until after birth. In post-natal patients with a clinical suspicion of cystic disease and positive family history, a genetic test should be performed on the affected parent(s) and, if positive, the identified variant(s) verified in the child, while in the absence of family history, genetic testing should be performed in the child (Fig. 4).

Taken together, these results indicate that early identification of pediatric patients with cystic kidney disease may result in their correct management and follow-up and eventually in targeted therapies to slow down disease progression.

## Supplementary Information

Below is the link to the electronic supplementary material.

Graphical Abstract (PPTX 494 KB)

## Data Availability

For data inquiries, please contact Tiziana Vaisitti (tiziana.vaisitti@unito.it).
