# Supplementary material for: Matching clinical and genetic data in pediatric patients at risk of developing cystic kidney disease
Source: Pediatr Nephrol. 2024 Oct 10;40(3):743–53. doi: 10.1007/s00467-024-06548-6 (PMC11747002; doi:10.1007/s00467-024-06548-6)
Supplement: Supplementary file 1 — Graphical Abstract (PPTX 494 KB) [file 467_2024_6548_MOESM1_ESM.pptx]

## Slide 1
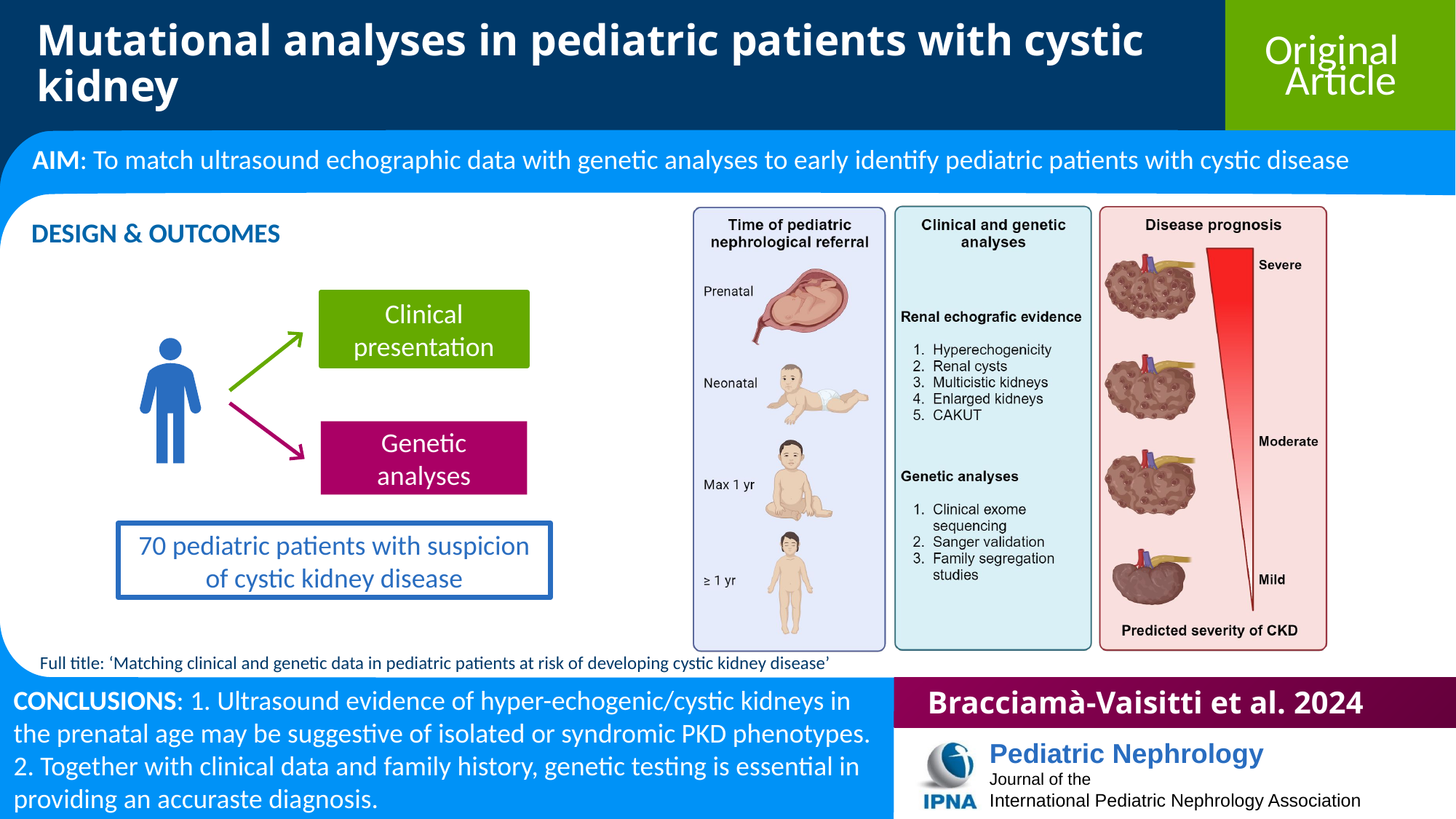

Mutational analyses in pediatric patients with cystic kidney
AIM: To match ultrasound echographic data with genetic analyses to early identify pediatric patients with cystic disease
DESIGN & OUTCOMES
Clinical presentation
Genetic analyses
70 pediatric patients with suspicion of cystic kidney disease
Full title: ‘Matching clinical and genetic data in pediatric patients at risk of developing cystic kidney disease’
CONCLUSIONS: 1. Ultrasound evidence of hyper-echogenic/cystic kidneys in the prenatal age may be suggestive of isolated or syndromic PKD phenotypes.
2. Together with clinical data and family history, genetic testing is essential in providing an accuraste diagnosis.
Bracciamà-Vaisitti et al. 2024
